# Indoor 3-D Localization Based on Received Signal Strength Difference and Factor Graph for Unknown Radio Transmitter

**DOI:** 10.3390/s19020338

**Published:** 2019-01-16

**Authors:** Liyang Zhang, Taihang Du, Chundong Jiang

**Affiliations:** 1 School of Artificial Intelligence, Hebei University of Technology, Tianjin 300130, China; thdu@hebut.edu.cn (T.D.); jchd@hebut.edu.cn (C.J.); 2 Control Engineering Research Center of Hebei, Tianjin 300130, China

**Keywords:** received signal strength difference (RSSD), radio transmitter, 3-D localization, factor graph (FG), sum-product algorithm

## Abstract

Accurate localization of the radio transmitter is an important work in radio management. Previous research is more focused on two-dimensional (2-D) scenarios, but the localization of an unknown radio transmitter under three-dimensional (3-D) scenarios has more practical significance. In this paper, we propose a novel 3-D localization algorithm with received signal strength difference (RSSD) information and factor graph (FG), which is suitable for both line-of-sight (LOS) and non-line-of-sight (NLOS) condition. Considering the stochastic properties of measurement errors caused by the indoor environment, RSSD measurements are processed with mean and variance in the form of Gaussian distribution in the FG framework. A new 3-D RSSD-based FG model is constructed with the relationship between RSSD and location coordinates by local linearization technique. The soft-information computation and iterative process of the proposed model are derived by using the sum-product algorithm. In addition, the impacts of different grid distances and number of signal receivers on positioning accuracy are explored. Finally, the performance of our proposed approach is experimentally evaluated in a real scenario. The results show that the positioning performance of the proposed algorithm is not only superior to the k-nearest neighbors (kNN) algorithm and least square (LS) algorithm, but also it can achieve a mean localization error as low as 1.15 m. Our proposed scheme provides a good solution for the accurate detection of an unknown radio transmitter under indoor 3-D space and has a good application prospect.

## 1. Introduction

The development of wireless network, mobile computing, pervasive computing, and other technologies makes location-based services and applications increasingly popular. Compared with two-dimensional (2-D) localization, simple plane positioning cannot meet the requirements of location services, and 3-D spatial localization has more practical application prospects. In contrast to the conventional positioning signal receiver, this paper aims to solve the three-dimensional (3-D) positioning issue of the unknown radio transmitter (such as illegal radio and pseudo base station) whose transmitting power and frequency are unknown. Therefore, achieving the precise localization of an unknown radio transmitter is a challenging task in radio management. Moreover, it is of great significance to strengthen the monitoring of radio spectrum resources, protect the interests of legitimate users and combat the occupation of illegal signal resources.

Currently, a variety of positioning techniques have been developed based on the measurements from the signal receiver, which is denoted as access point (AP). The measurement information mainly includes time of arrival (TOA) [1], time difference of arrival (TDOA) [2], angle-of-arrival (AOA) [3], received signal strength (RSS) [4,5], and hybrid of them [6,7,8]. Among them, RSS is widely used for its advantages of simple positioning algorithm, low cost, low power consumption and no additional hardware. The conventional RSS-based fingerprint positioning technique can be roughly divided into two phases: the off-line training phase and the on-line positioning phase. In the off-line training phase, RSS measurements of different locations in the positioning area are sampled manually and stored with the corresponding location coordinates in the fingerprint database. In the on-line positioning phase, server matches the fingerprint information at the location of the positioning target with the fingerprint database and searches the location information corresponding to the fingerprint with the appropriate positioning algorithm as the location of the positioning target. Two typical RSS-based fingerprint positioning algorithms are RADAR [9] and LANDMARC [10] methods. The basic principle of the above two methods is to select *k* reference points using Euclidean distance in the form of RSS measurements. The process is denoted as k-nearest neighbors (kNN) algorithm [11]. The least square (LS) algorithm is another well-known approach with using the measurement information to estimate the geometric distance between the target and AP and to find the optimal location of the target by minimizing the sum of squares of errors [12]. However, the mentioned conventional algorithms do not fully consider the stochastic properties of measurement errors, and the Euclidean distance characterized by RSS cannot fully reflect the geometric distance. Therefore, the positioning performance cannot be effectively improved in complex indoor positioning scenario. An accurate localization framework by unsupervised fusion of an extended candidate location set (ECLS) was proposed to overcome the impact of changing positioning environment and model errors [13]. Nevertheless, the RSS-based positioning method is mainly used to solve the localization issue of signal receiver and cannot achieve the accurate localization of the unknown radio transmitter. Instead of using the RSS measurements, a positioning technique based on received signal strength difference (RSSD) is used to the localization of radio transmitter. While preserving all the advantages of RSS-based localization, it can significantly alleviate the passive dependence of localization on the radio transmitter, which could be defective, malicious, or uncooperative [14]. Besides, the RSSD-based parameter can also eliminate the influence of device differences on positioning accuracy caused by the difference between on-line positioning device and off-line database establishment device [15]. An efficient RSSD-based source localization technique was proposed for the case of unknown transmit power and uncertainty in the sensor locations [16]. A new minimax-semidefinite programming (minimax-SDP) method for the RSSD-based measurement model was recently developed which provides a significant improvement over other RSSD-based methods [17].

Among all positioning algorithms, the FG-based technique is famous for the low computational complexity and high positioning accuracy [18]. Many FG positioning methods based on different measurement information have been developed in recent years. The TOA-FG [19] technique used the time of signal propagation between the radio transmitter and APs to estimate the distance, but it requires the clocks of both to be synchronous. To be free from the limitation of clock synchronization between the radio transmitter and APs, TDOA-FG method was proposed in [20]. It should be noted that radio transmitter and APs also need to be synchronized with the clock of the reference node. However, both TOA-FG and AOA-FG techniques are not suitable for indoor positioning due to the lack of line-of-sight (LOS) scenario. The RSS-FG technique not only overcomes the requirement of perfect synchronization or time stamp but also adapts the LOS and non-line-of-sight (NLOS) positioning scenario [21]. Yet, the RSS-FG method has been proved to be unable to achieve the localization of the unknown radio transmitter since both transmitting frequency and power of the radio transmitter are unknown. Therefore, the RSSD-FG [22] technique was developed to realize the localization of the unknown radio transmitter successfully. However, the above researches only focus on positioning in a 2-D scenario. Due to the large number of unknown parameters, it is more difficult to achieve the localization in 3-D space, and there is no research on localization of an unknown radio transmitter using FG in 3-D scenario. Therefore, the realization of accurate localization of unknown radio transmitter using FG method in indoor 3-D space has a high application prospect.

In this paper, we first combine the RSSD-based fingerprint positioning method and FG technique to propose a 3-D RSSD-FG algorithm. A new 3-D RSSD-based FG model is constructed to express the RSSD information and location coordinates. At first, we use the RSSD fingerprint database in the off-line training phase to obtain the local linear relationship between RSSD information and corresponding location coordinates. In the proposed FG model, the stochastic properties of measurement errors are processed with Gaussian distribution, which is more reasonable to reflect the impact of indoor uncertain interference factors. Using the sum-product algorithm, the soft information can be calculated and constantly updated among the factor nodes and variable nodes. Then, the variable nodes of target location can be estimated after several iterations. To verify the correctness and feasibility of the proposed algorithm, we conduct the experiments in a real office environment on the first floor of the National Radio Monitoring Center (Beijing), where realistic measurements are performed. The experimental results show that the positioning accuracy of the proposed algorithm is higher than that of the KNN and LS algorithms in the case of different grid distances or different AP numbers, and the probability of positioning error within 1.5 m can reach over 70%. It can be concluded that our proposed algorithm can significantly improve the positioning accuracy compared with the conventional positioning algorithms.

## 2. The System and Principle

### 2.1. Positioning System for the Unknown Radio Transmitter

**Figure 1:**
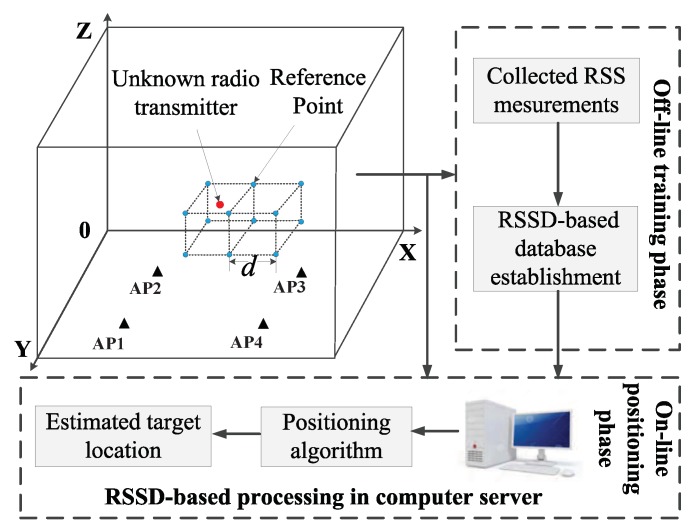
Basic structure of the RSSD-based fingerprint positioning system with four APs and an unknown radio transmitter.

A typical RSSD-based indoor fingerprint positioning system for the radio transmitter is described in Figure 1. The positioning area is divided into several cubes with the vertices set as the reference points. Then, four APs are placed in the positioning area to collect the RSS measurements from the radio transmitter. The length between two adjacent reference points is defined as grid distance (*d*). In RSSD-based database establishment phase, we select a known radio transmitter to traverse the obtained reference points and record the collected RSS measurements information from the APs. After that, the subtraction of RSS measurements from two different APs is stored in the fingerprint database along with the location of reference point. In our research, the RSSD-based database is used because it has adaptability to various unknown radio transmitters [14]. Thus, we only need to establish the off-line database once, which can greatly reduce the workload of constructing different databases to match with different radio transmitters.

When an unknown radio transmitter enters the positioning area, the APs collect the real-time RSS measurements and RSSD information will be reported to the computer server. At last, the estimated location of positioning target can be obtained by the specific algorithm. The detailed process of our proposed algorithm will be introduced in the next section.

### 2.2. Factor Graph and Sum-Product Algorithm

**Figure 2:**
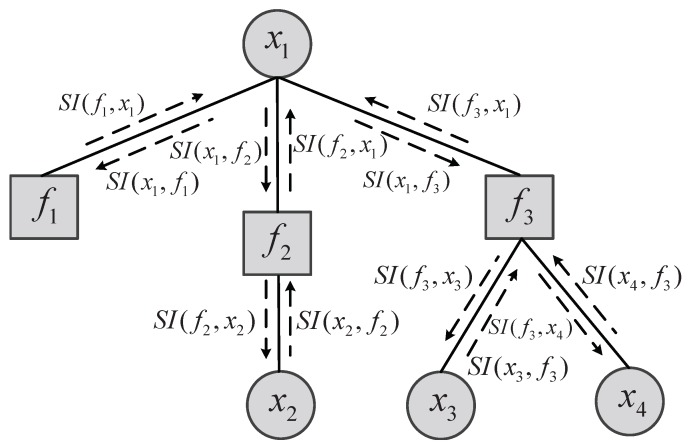
Fragment of a FG showing the soft-information transported rules of sum-product algorithm.

In this section, we first introduce the basic theoretical knowledge of the FG model and sum-product algorithm [23]. A FG is a bidirectional graph that can decompose complex global functions into the product of several local functions with fewer variables. A generic FG usually consists of the variable node and the factor node as shown in Figure 2.

The variable nodes and factor nodes are indicated by circles and squares, respectively. As shown in Figure 2, the soft-information transported among the variable nodes and factor nodes represents the statistical properties of the estimated variables and measurement errors in the form of a Gaussian probability density function. In this paper, we define SI(a,b) as the soft-information transported from node a to node b, which follows a Gaussian distribution ($SI\left(a,b\right)∼N\left(m_{a,b},\sigma_{a,b}^{2}\right)$) with mean $m_{a,b}$ and variance $\sigma_{a,b}^{2}$. The factor node $f_{i}\left(\cdot \right)$ represents the local mathematical relationship associated with its connected variable node $x_{i}$. The FG shown in Figure 2 is the description of $f\left(x_{1},x_{2},x_{3},x_{4}\right)=f_{1}\left(x_{1}\right)\cdot f_{2}\left(x_{1},x_{2}\right)\cdot f_{3}\left(x_{1},x_{3},x_{4}\right)$ expressed by variable nodes $\left(x_{1},x_{2},x_{3},x_{4}\right)$ and factor nodes $\left(f_{1},f_{2},f_{3}\right)$. Thus, a complex problem described by variables in the FG can be solved by soft-information iterative process between the variable nodes and the factor nodes. The soft information transmitted between factor nodes and variable nodes can be obtained by using the sum-product algorithm that collects the soft information expressed by the local function in the form of product. Moreover, each piece of soft information from the variable node to the factor node expresses the stochastic properties of the associated variable nodes. This kind of soft information is composed of the product of all soft information transmitted from other factor nodes to the variable nod. For example, the soft information $SI(x_{1},f_{1})$ shown in Figure 2 can be obtained by

(1) $$SI\left(x_{1},f_{1}\right)=SI\left(f_{2},x_{1}\right)\cdot SI\left(f_{3},x_{1}\right).$$

Since the product of several independent gaussian distributions still follows the gaussian distribution, the mean and variance of soft information $SI(x_{1},f_{1})$ can be calculated by

(2) $$m_{x_{1},f_{1}}=\sigma_{x_{1},f_{1}}^{2}\cdot \sum_{t=2}^{3}\frac{m_{x_{t},f_{t}}}{\sigma_{x_{t},f_{t}}^{2}},$$

and

(3) $$\sigma_{x_{1},f_{1}}^{2}=\sum_{t=2}^{3}\frac{1}{\sigma_{x_{t},f_{t}}^{2}}.$$

The soft information transported from a factor node to a variable node can be obtained by the product of the local function related to the factor node and each piece of the soft information passing from other variable nodes. However, it is important to note that the variable nodes passing the soft information to the factor node does not contain the variable node receiving soft information. Taking $SI(f_{3},x_{1})$ for an example, the soft information can be expressed by

(4) $$SI\left(f_{3},x_{1}\right)=\int_{x_{3}}\int_{x_{4}}f_{3}\left(x_{1},x_{3},x_{4}\right)\cdot SI\left(x_{3},f_{3}\right)\cdot SI\left(x_{4},f_{3}\right)dx_{3}dx_{4},$$

where $f_{3}\left(x_{1},x_{3},x_{4}\right)$ is the local function associated with the variable nodes $x_{1}$, $x_{3}$, and $x_{4}$. With the above calculation rules, all the soft information between variable nodes and factor nodes in the FG can be obtained. When the soft information converges through some iterative processes, the estimate value of each variable can be obtained by the product of all the soft information transported from factor nodes to the variable node. All soft information passed to the variable node $x_{1}$ can be expressed by

(5) $$SI\left(x_{1}\right)=SI\left(f_{1},x_{1}\right)\cdot SI\left(f_{2},x_{1}\right)\cdot SI\left(f_{3},x_{1}\right).$$

## 3. The Proposed Algorithm

### 3.1. 3-D RSSD-Based FG Model

**Figure 3:**
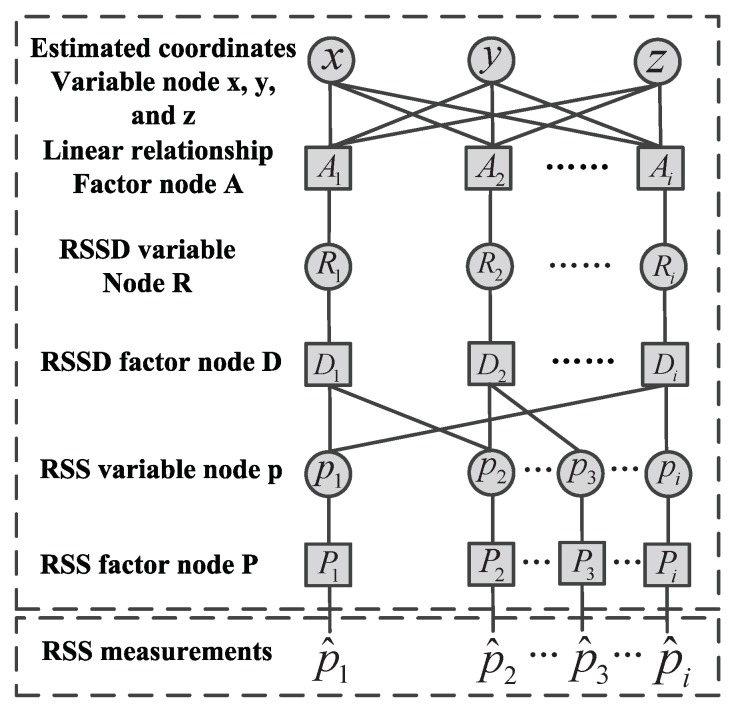
The proposed 3-D RSSD-based factor graph model.

In this section, we construct a 3-D RSSD-based FG model to estimate the location of an unknown radio transmitter as shown in Figure 3. The proposed 3-D RSSD-based FG model also consists of the variable nodes $\left(x,y,z,R_{i},p_{i}\right)$ and the factor nodes $\left(A_{i},D_{i},P_{i}\right)$, where *i* is the index number of AP (i=1,2,…,N).

First, different APs of the positioning area take the acquired RSS measurements as the input parameter of the proposed FG model. Thus, the reported RSS measurement from *i*-AP can be expressed as

(6) $${\hat{p}}_{w,i}={\tilde{p}}_{w,i}+e_{i},$$

where ${\tilde{p}}_{w,i}$ is the error free measurement from *i*-th AP in unit of watts, $e_{i}$ represents the measurement error caused by the interference factors of the positioning environment that follows the Gaussian distribution $\left(e_{i}∼N\left(0,\sigma_{i}^{2}\right)\right)$ as done in [24,25]. The error caused by the NLOS components can be included to the variance of the measurement error as shown in [26]. In this way, our method is also applicable to NLOS scenario. To better reflect the local linearity feature in the proposed algorithm, the measured RSS ${\hat{p}}_{w,i}$ is expressed in the form of logarithmic scale $\left({\hat{p}}_{i}=10\cdot \log_{10}\left({\tilde{p}}_{w,i}+e_{i}\right)\right)$. In addition, the logarithmic RSS has also been demonstrated to have an approximate Gaussian distribution [21]. The factor node $P_{i}$ expresses the Gaussian statistical distribution relationship of the mean $\left({\tilde{p}}_{i}\right)$ and variance $\left(\sigma_{p_{i}}^{2}\right)$ generated by logarithmic RSS measurement. Therefore, the variable node $p_{i}$ follows the Gaussian distribution as $\left(p_{i}∼N\left({\tilde{p}}_{i},\sigma_{p_{i}}^{2}\right)\right)$. Then, the factor node $D_{i}$ represents the relationship of logarithmic RSS subtraction between two different APs. The combination of the two different APs can be “12, 23, …, *i*1”. For example, the variable node $R_{1}$ can be obtained by

(7) $$R_{1}=p_{1}-p_{2}.$$

Thus, other different AP combinations can be obtained in the same way such as $R_{2}=p_{2}-p_{3}$, $R_{3}=p_{3}-p_{4}$, …, and $R_{i}=p_{i}-p_{1}$, where *i* is the AP combination serial number. According to the path loss propagation model, the RSS received by AP is related to the geometric distance between the radio transmitter and AP. Then, each location coordinate of the positioning area corresponds to a specific RSSD value of an AP combination. Thus, the relationship between logarithmic RSSD (r) and location coordinates (x,y,z) can be modeled as

(8) $$k_{x}\cdot x+k_{y}\cdot y+k_{z}\cdot z+k_{r}\cdot r=c,$$

where $k_{x}$, $k_{y}$, $k_{z}$, and $k_{r}$ are coefficients of the equation, *c* is a nonzero constant. To obtain the coefficients in Equation (8), the sub-localization region in which the target is located should be first determined. This paper use the pattern-recognition method [21] to select five reference points. Since the logarithmic RSSD measurements and the location of the five selected reference points have been obtained, five formulas like (8) in matrix form for *i*-th AP combination are given by

(9) B·K=C,

where

(10) $$B=\left[\begin{array}{cccc} x_{1} & y_{1} & z_{1} & {\tilde{R}}_{i,1}\\ x_{2} & y_{2} & z_{2} & {\tilde{R}}_{i,2}\\ x_{3} & y_{3} & z_{3} & {\tilde{R}}_{i,3}\\ \begin{array}{c} x_{4}\\ x_{5} \end{array} & \begin{array}{c} y_{4}\\ y_{5} \end{array} & \begin{array}{c} z_{4}\\ z_{5} \end{array} & \begin{array}{c} {\tilde{R}}_{i,4}\\ {\tilde{R}}_{i,5} \end{array} \end{array}\right],K=\left[\begin{array}{c} k_{x,i}\\ k_{y,i}\\ k_{z,i}\\ k_{r,i} \end{array}\right],C=\left[\begin{array}{c} 1\\ 1\\ 1\\ \begin{array}{c} 1\\ 1 \end{array} \end{array}\right].$$

In Equation (10), $k_{x,i}$, $k_{y,i}$, $k_{z,i}$, and $k_{r,i}$ are the coefficients of the equation related to *i*-th AP combination. $\left(x_{j},y_{j}\right)$ is the location coordinate of *j*-th reference point. where j=1,2,3,4,5. ${\tilde{R}}_{i,j}$ is the mean logarithmic RSSD measurement of *j*-th reference point from *i*-th AP combination. According to lest square algorithm, the coefficient matrix K can be calculated by $K={\left(B^{T}\cdot B\right)}^{-1}\cdot B^{T}\cdot C$, where ${\left(\cdot \right)}^{T}$ and ${\left(\cdot \right)}^{-1}$ are defined as the matrix transpose and matrix inverse, respectively. In this manner, the relationship between location coordinates and *i*-th logarithmic RSSD within the choosing the sub-localization region is obtained. Due to the sub-localization region located at the target is known, the logarithmic RSSD variable *r* can be replaced with the variable of the target logarithmic RSSD $R_{i}$. Then, the local linear relationship represented by factor node $A_{i}$ between logarithmic RSSD measurement of *i*-th AP combination and location coordinate of the target is given by

(11) $$k_{x,i}x+k_{y,i}y+k_{z,i}z+k_{r,i}R_{i}=1.$$

Thus, the other local linear relationships corresponding to different AP combinations can be obtained in the same way. After the local function relationships of all factor nodes and variable nodes are established, factor nodes use these local functions to transport the soft information from variable nodes. Finally, the soft information of the target variable nodes (x,y,z) are obtained by the product of all soft information transported from the connected factor nodes. In this way, the soft information of the target variable nodes will be updated continuously with the iterative process of the soft information transferred between the factor node and the variable node. The iterative process and calculation of all soft information will be introduced in the next subsection.

### 3.2. Soft Information Calculation and Iterative Process

In this subsection, we introduce the detailed description of calculating the soft information and the iterative process of the proposed FG model. First, the soft information transported from variable nodes *x*, *y*, and *z* to factor node $A_{i}$ can be calculated with the product of all the soft information transported from the rest of the factor nodes to the variable node. Then, the soft information $SI(x,A_{i})$, $SI(y,A_{i})$, and $SI(z,A_{i})$ can be given by

(12) $$SI\left(x,A_{i}\right)=\prod_{t\neq i}^{N}\left(A_{t},x\right),$$

(13) $$SI\left(y,A_{i}\right)=\prod_{t\neq i}^{N}\left(A_{t},y\right),$$

and

(14) $$SI\left(z,A_{i}\right)=\prod_{t\neq i}^{N}\left(A_{t},z\right).$$

Here, it should be noted that the product of some independent Gaussian probability density function is still a Gaussian probability density function [27]. Therefore, taking $SI(x,A_{i})$ for an example, the mean and variance are given by

(15) $$m_{x,A_{i}}=\sigma_{{}_{x,A_{i}}}^{2}\left(\sum_{t\neq i}^{N}\frac{m_{A_{t},x}}{\sigma_{A_{t},x}^{2}}\right),\sigma_{x,A_{i}}^{2}=1/\left(\sum_{t\neq i}^{N}\frac{1}{\sigma_{A_{t},x}^{2}}\right).$$

In the same way, the soft information $SI(x,A_{i})$ and $SI(y,A_{i})$ can also be obtained. Next, factor node $A_{i}$ combine with the information coming from the variable nodes *x*, *y*, and *z* to update the new soft information related to *x*, *y*, and *z* under the local linear function described in (11). According to Equation (11), the mean and variance of soft information $SI(A_{i},x)$ can be obtained by

(16) $$m_{A_{i},x}=\left(1-k_{y,i}m_{y,A_{i}}-k_{z,i}m_{z,A_{i}}-k_{r,i}m_{R_{i},A_{i}}\right)/k_{x,i}$$

and

(17) $$\sigma_{A_{i},x}^{2}=\left(k_{y,i}^{2}\sigma_{y,A_{i}}^{2}+k_{z,i}^{2}\sigma_{z,A_{i}}^{2}+k_{r,i}^{2}\sigma_{R_{i},A_{i}}^{2}\right)/k_{x,i}^{2}.$$

Thus, $SI(A_{i},y)$ and $SI(A_{i},z)$ can also be obtained in this way. The soft information transported from variable node $R_{i}$ to factor $A_{i}$ is equal to factor node $D_{i}$ to variable node $R_{i}$, where $m_{R_{i},A_{i}}=m_{D_{i},R_{i}}$ and $\sigma_{R_{i},A_{i}}^{2}=\sigma_{D_{i},R_{i}}^{2}$. According to Equation (7), the mean and variance of soft information $SI(D_{i},R_{i})$ is expressed by

(18) $$m_{D_{i},R_{i}}=m_{p_{i},D_{i}}-m_{p_{1},D_{i}}$$

and

(19) $$\sigma_{D_{i},R_{i}}^{2}=\sigma_{p_{i},D_{i}}^{2}+\sigma_{p_{1},D_{i}}^{2}.$$

The factor nodes $P_{1}$ and $P_{i}$ directly transport the soft information to node $D_{i}$, where $m_{p_{1},D_{i}}=m_{P_{1},p_{1}}$, $\sigma_{p_{1},D_{i}}^{2}=\sigma_{P_{1},p_{1}}^{2}$ and $m_{p_{i},D_{i}}=m_{P_{i},p_{i}}$, $\sigma_{p_{i},D_{i}}^{2}=\sigma_{P_{i},p_{i}}^{2}$. Then, the mean and variance of $SI(P_{1},p_{1})$ and $SI(P_{i},p_{i})$ can be directly obtained by the RSS measurements. Finally, the soft information of the target estimated location can be updated by combining all the soft information from all the factor node $A_{i}$ to the variable node *x*, *y*, and *z*, respectively. The calculation formulas of SI(x), SI(y), and SI(z) can be calculated by

(20) $$m_{x}=\sigma_{x}^{2}\cdot \left(\sum_{i=1}^{N}\frac{m_{A_{i},x}}{\sigma_{A_{i},x}^{2}}\right),\sigma_{x}^{2}=1/\left(\sum_{i=1}^{N}\frac{1}{\sigma_{A_{i},x}^{2}}\right),$$

(21) $$m_{y}=\sigma_{y}^{2}\cdot \left(\sum_{i=1}^{N}\frac{m_{A_{i},y}}{\sigma_{A_{i},y}^{2}}\right),\sigma_{y}^{2}=1/\left(\sum_{i=1}^{N}\frac{1}{\sigma_{A_{i},y}^{2}}\right),$$

and

(22) $$m_{z}=\sigma_{z}^{2}\cdot \left(\sum_{i=1}^{N}\frac{m_{A_{i},z}}{\sigma_{A_{i},z}^{2}}\right),\sigma_{z}^{2}=1/\left(\sum_{i=1}^{N}\frac{1}{\sigma_{A_{i},z}^{2}}\right).$$

**Figure 4:**
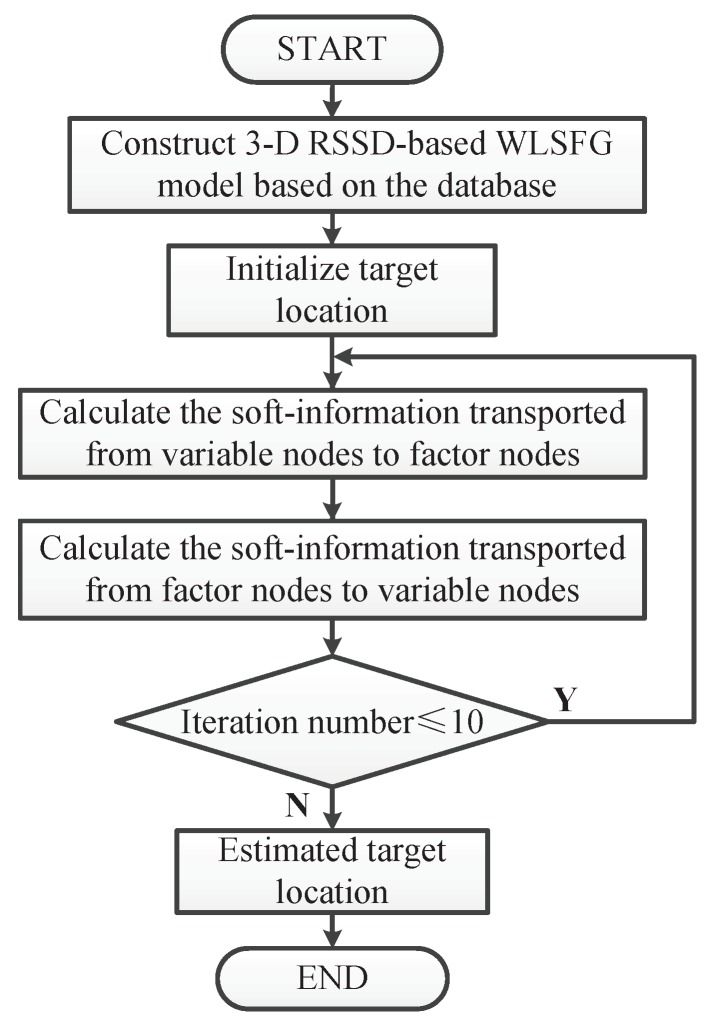
Flow chart of the proposed algorithm.

With the formulas from (20)–(22), the estimated location coordinates of the target are $m_{x}$, $m_{y}$, and $m_{z}$, respectively. As mentioned above, all soft information can be calculated by the sum-product algorithm, and the whole iterative process will be repeated repeatedly. Figure 4 is the flow chart of the proposed algorithm. To facilitate understanding, we summarized the detailed iteration process as shown in Table 1. According to the simulation experience, the soft information will converge with the number of iterations reaching 10. Although the mathematical proof of convergence is not given in this paper, the experimental result verifies it. This may be due to the proposed method considering the stochastic properties of measurement errors. In addition, the initialization of the target location does not have a critical impact on convergence and can be set to arbitrary value. The iterative process will not stop until the iteration number reaches the set value.

It is obviously obtained from Table 1 that the proposed method only requires simple arithmetic operations on each node. As shown in [21], the computational complexity of the conventional RSS-FG algorithm is linearly proportional to *N*. Compared with RSS-FG algorithm, the proposed algorithm only adds subtraction operation and the increase of dimension does not change the order of the local linear relationship, so the computational complexity of the proposed algorithm is also linearly proportional to *N*. Therefore, the proposed 3-D RSSD-based FG algorithm also enjoys low computational complexity the same as RSS-FG algorithm.

## 4. Results and Discussions

### 4.1. Simulation Analysis

The computer simulations were conducted on the platform of MATLAB2014a to demonstrate the correctness of the proposed algorithm. The dimensions of the simulation space are 100 m, 100 m and 100 m in the form of length, width, and height, respectively. A well-known logarithmic shadowing model [28] applied to generate the RSS measurements for the off-line database construction and the on-line positioning target, which is given by

(23) $$P\left(d_{i,j}\right)=P\left(d_{0}\right)-10\cdot \alpha \cdot \log_{10}\left(\frac{d_{i,j}}{d_{0}}\right)+\chi ,$$

where $P(d_{i,j})$ is the RSS of *j*-th reference point from *i*-th AP, $d_{i,j}$ is the distance between *j*-th reference point and *i*-th AP, $P(d_{0})$ is the RSS in decibel at the reference $d_{0}$, α is path loss exponent, $d_{0}$ is the reference distance, and χ represents the variance of RSS measurement following zero-mean Gaussian distribution ($\chi ∼N(0,\sigma_{\chi }^{2})$). Then, the random RSS measurement ${\hat{p}}_{i,j}$ can be considered as Gaussian distribution (${\hat{p}}_{i,j}∼N\left(P\left(d_{0}\right)-10\cdot \alpha \cdot \log_{10}\left(\frac{d_{i,j}}{d_{0}}\right),\sigma_{\chi }^{2}\right)$). The value of the simulation parameters of Formula (23) are as follows: $d_{0}$ = 1 m, $P(d_{0})$ = 10 dB, $\sigma_{\chi }$ = 5.2 dB, and α = 1.8, and the on-line real-time RSS measurement of the target can be generated as done in [21].

**Figure 5:**
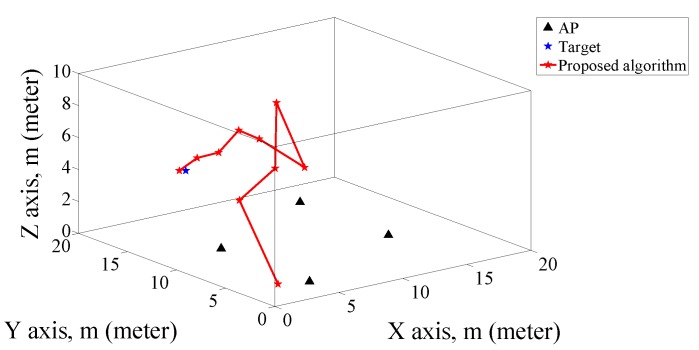
Positioning trajectory of the proposed algorithm with 10 iterations, 2 m grid distance, target location at (3, 13, 5) m and target initialization at (1, 1, 1) m.

First, we use four APs and 2 m grid distance, and 100 target locations were randomly selected in the positioning area. The location coordinates of the four APs marked with black solid triangle are (5, 3, 0) m, (15, 8, 0) m, (5, 12, 0) m and (15, 17, 0) m, respectively. The detailed process of localization trajectory of the proposed algorithm is as shown in Figure 5. It is clear from the figure that the estimated location of the target is close to the actual location after the number of iterations reaching 10, where the target location is at (3, 13, 5) m and the initial location is set at (1, 1, 1) m.

**Figure 6:**
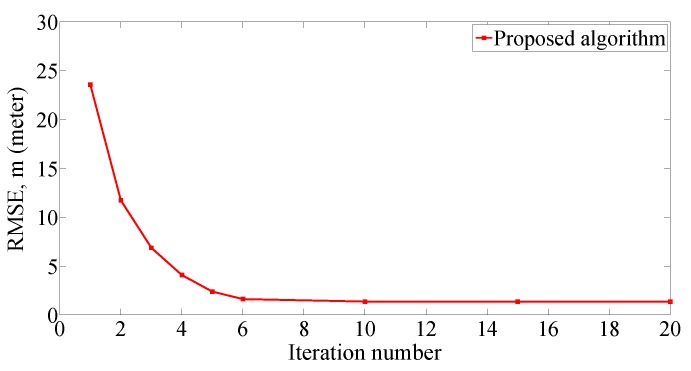
RMSE of proposed algorithm with the iteration number changing from 1 to 20, four APs and 2 m grid distance.

Figure 6 shows that the root mean square error (RMSE) of the proposed technique converges as the iteration number increases. The RMSE tends to be stable at 1.45 m with the iteration number drawing near to 10. Therefore, it can be seen that the proposed algorithm has the characteristic of rapid convergence.

**Figure 7:**
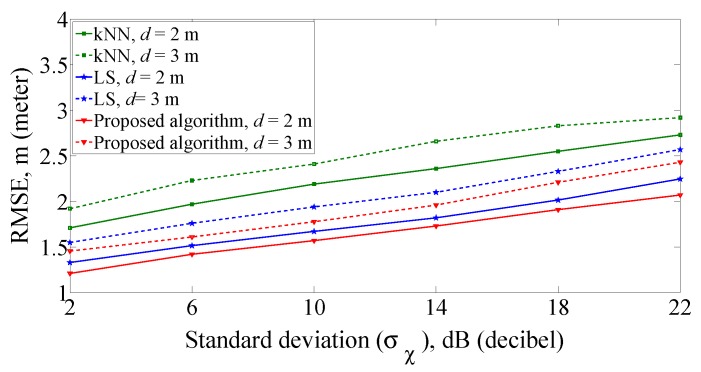
RMSE comparison with different grid distances.

Next, the RMSE of proposed algorithm is compared with the kNN (k = 4) algorithm and LS algorithm in the case of different grid distances and number of APs, when the standard deviation of RSS measurement is different. First, we compare the positioning accuracy of the three algorithms with different grid distances when the number of APs is four. As can be seen from the simulation results in Figure 7, the proposed algorithm is more accurate than the kNN algorithm and the LS algorithm under corresponding grid distance and lower grid distance can effectively improve the positioning accuracy. Taking $\sigma_{\chi }$ = 6 dB and *d* = 2 m for an example, the RMSE of the proposed algorithms is 1.41 m. The RMSE for other two algorithms are 1.55 m for LS algorithm and 1.82 m for kNN algorithm.

**Figure 8:**
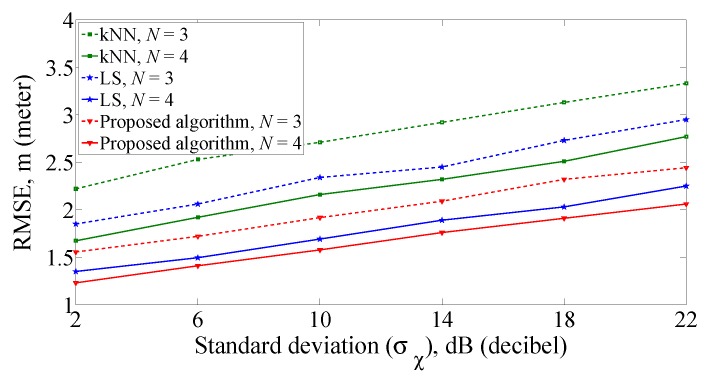
RMSE of position errors with different number of APs.

Then, we explore the impact of different AP numbers on positioning performance when the grid distance is 2 m. As shown in Figure 8, the RMSE of three algorithms decrease as the number of APs increase. Compared with the kNN algorithm and LS algorithm, RMSE of the proposed algorithm is the smallest and the positioning accuracy is the highest in the case of the corresponding number of APs. For example, when $\sigma_{\chi }$ = 6 dB and *N* = 3, the RMSE of kNN algorithm and LS algorithm are 2.53 m and 2.06 m, respectively. In comparison, RMSE of the proposed algorithm is 1.72 m. Moreover, the positioning accuracy of using three APs is higher than that of kNN algorithm using four APs. Considering the requirement of positioning accuracy and cost, four APs are used in the following experiments to conduct positioning research.

Even considering the stochastic properties of RSS measurement error, the above results prove that the proposed algorithm can obtain higher positioning accuracy than that using the conventional deterministic algorithms. Therefore, it has better adaptability to the complex indoor environment in practical application.

### 4.2. Experimental Results

**Figure 9:**
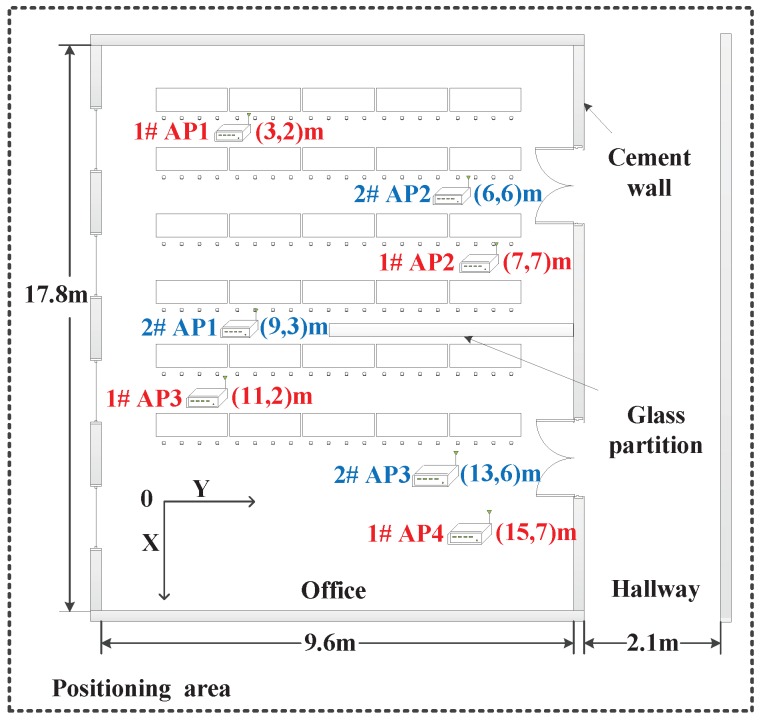
Plane layout of the test field with 17.8 m length and 11.7 m width and the height of test space is 4.8 m.

To verify the performance of the proposed algorithm, experimental tests were carried out on the first floor of the National Radio Monitoring Center, Beijing. The test field consists of two parts, an office and its adjacent hallway. To embody the internal structure of the positioning area more intuitively, Figure 9 shows the plane layout of the test field. The areas of the office and hallway are 17.8 m × 9.6 m and 17.8 m × 2.1 m, respectively, and the height of the whole positioning area is 4.8 m. The space size of the entire positioning area is length 17.8 m × width 11.7 m × height 4.8 m. The number and layout of APs are selected with two types, which are marked with “1 #” and “2 #” as shown in Figure 9. The location coordinates of four APs are (3, 2, 0.8) m, (7, 7, 0.8) m, (11, 2, 0.8) m, and (15, 7, 0.8) m, respectively. The location coordinates of three APs are (9, 3, 0.8) m, (6, 6, 0.8) m, and (13, 6, 0.8) m, respectively. There is a glass partition in the middle of the office, which length is 3.3 m. In addition, there are four windows on one wall of the office and two doors on the wall near the hallway. The main items in the office are desks, chairs, computers, and other office supplies. The staff are free to enter and leave frequently during the whole experiment. Since the positioning area includes an office with glass partition and its adjacent hallway, the experimental scenario has the characteristics of LOS and NLOS.

The grid distances used to construct the fingerprint database are selected with 1.5 m and 2 m, respectively. When using the minimum grid distance (1.5 m) in our test, it takes about two hours to construct the fingerprint database. The signal receivers are used with SA44B model from Signal Hound Co. Ltd. The radio transmitter used for fingerprint database construction and on-line positioning test is TFG6300 model with adjustable “frequency/strength” manufactured by SUING Co. Ltd. To prove that the proposed algorithm is not affected by the frequency and strength of the off-line fingerprint database, the off-line database and on-line positioning test of “frequency/strength” are selected with “1 GHz/20 dB” and “300 MHz/13 dB”, respectively. In the experiment, fifty target locations are randomly selected in the test field for on-line positioning test, and the height of these targets ranges from 0.5 m to 4.3 m. To lower the influence of personnel movement, indoor items and measuring equipment error on the accuracy of ${\tilde{p}}_{i}$, the error can be reduced by the accurate measurement equipment and averaging a lot of sample data and the number of RSS samples in the experiment is 100.

Next, the mean position error and cumulative distribution function (CDF) are used as two key indicators to compare the positioning performance among the proposed algorithm, kNN algorithm, and LS algorithm. The mean position error expresses the average value of all the position errors between the estimated targets and the real targets. The CDF expressed as a percentage represents the number of test targets within a certain range of position error. First, mean position errors of the three algorithms with four APs are compared under different grid distances, which are 1.5 m and 2 m as shown in Table 2. It is observed that the mean position errors of kNN algorithm and LS algorithm are 1.41 m and 1.37 m, respectively. The mean position error of the proposed algorithm is 1.12 m in comparison. However, the positioning accuracy decreases with the increase of grid distance. As the grid distance increases to 2 m, the mean position errors for the three algorithms are 1.25 m for the proposed algorithm, 1.46 m for LS algorithm, and 1.62 m for kNN algorithm. For the proposed algorithm, this is because the larger grid distance leads to less RSS information collected by the off-line database and the local linear relationship composed of selected reference points becomes rough.

**Figure 10:**
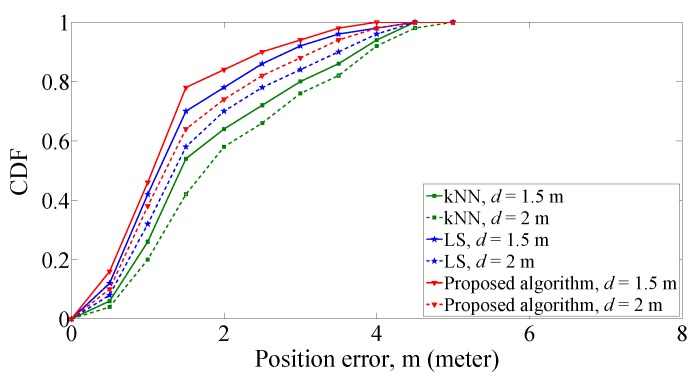
CDF of position errors with different grid distances.

The CDF comparison among the different algorithms is as shown in Figure 10. It is shown that the number of qualified test targets of the proposed algorithm within different position errors is larger than that of the other two algorithms under the corresponding grid distance. Considering the position error within 1.5 m, the CDF for each algorithm is 54% for kNN, 68 percent for LS, and 78% for the proposed algorithm as the grid distance is 1.5 m. When the grid distance increases to 2 m, the CDF of proposed algorithm, kNN algorithm, and LS algorithm is 64%, 42%, and 58% respectively. The results demonstrate that the smaller grid distance can improve the positioning accuracy. In the case of different grid distances, the positioning performance of proposed algorithm is superior to the other two algorithms.

Next, the effect of the number of AP on the positioning accuracy was explored through the experiments. The grid distance is selected with 1.5 m to evaluate the positioning performance of the three algorithms when the number of APs are three and four, respectively. The comparison of the mean location errors among different algorithms with 1.5 m grid distance is as shown in Table 3. When the number of AP is three, the mean position error of the proposed algorithm is 1.41 m. The kNN algorithm and LS algorithm are 1.83 m and 1.58 m, respectively. As the number of AP increases to four, the mean position errors for each algorithm is 1.65 m for kNN algorithm, 1.37 m for LS algorithm and 1.15 m for the proposed algorithm. The above experiment results demonstrate that the increase of the number of AP can improve the positioning accuracy. In the case of different number of AP, the positioning performance of proposed algorithm is superior to the other two algorithms.

**Figure 11:**
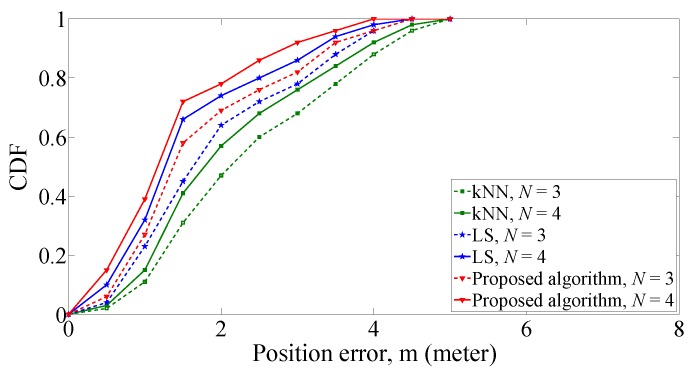
CDF of position errors with different number of APs.

Figure 11 shows that the CDF comparison of position errors under different number of APs. With 1.5 m grid distance and three APs, the CDF of proposed algorithm within 1.5 m position error is 58%. The kNN algorithm and LS algorithm are 45% and 31%, respectively. When the number of AP is four, the CDF of proposed algorithm increases to 72%. The CDF for other two algorithms is 41% for kNN algorithm and 66% for LS algorithm.

The above experimental results show that the proposed algorithm has a higher positioning accuracy than that of kNN algorithm and LS algorithm no matter under different grid distances or different number of APs.

## 5. Conclusions

In this paper, we have proposed a new 3-D indoor localization algorithm to achieve more accurate detection of an unknown radio transmitter. A 3-D RSSD-bsed FG model was constructed based on the local linear relationship between RSSD and location coordinates. Using the Gaussian assumption of measurement errors and taking the stochastic properties of measurement error into account can better reflect the various impact factors of complex indoor environment than the conventional deterministic algorithms. The soft information transported between factor nodes and variable nodes in the proposed FG can be obtained by sum-product algorithm. The location variable of the target can be calculated by a certain number of exchanging soft information, and the proposed method enjoys fast convergence. In addition, the positioning performance was explored under different distances and different number of APs through the simulations. After that, we implemented our proposed scheme in a real 3-D scenario to verify the feasibility, and the mean localization error can be approximately 1.2 m. Compared with the kNN algorithm and LS algorithm, the proposed algorithm effectively improves the positioning accuracy by about 25% and 15%, respectively. The experiment results show that the proposed method has better positioning performance than the other two algorithms no matter under the different grid distances and number of APs. The impact of AP’s layout on positioning accuracy and localization of the multi-targets will be involved in our future researches.

## Figures and Tables

**Table 1:** The operations of each node in Figure 3.

Node	Input (Mean, Variance)	Output (Mean, Variance)
$P_{i}$	$\left({\hat{p}}_{i},0\right)$	$\left(m_{P_{i},p_{i}},\sigma_{P_{i},p_{i}}^{2}\right)$
$p_{i}$	$\left(m_{P_{i},p_{i}},\sigma_{P_{i},p_{i}}^{2}\right)$	$\left(m_{p_{i},D_{i}},\sigma_{p_{i},D_{i}}^{2}\right)$
$D_{i}$	$\left(m_{p_{i},D_{i}},\sigma_{p_{i},D_{i}}^{2}\right)$, $\left(m_{p_{1},D_{i}},\sigma_{p_{1},D_{i}}^{2}\right)$	$m_{D_{i},R_{i}}=m_{p_{i},D_{i}}-m_{p_{1},D_{i}}$, $\sigma_{D_{i},R_{i}}^{2}=\sigma_{p_{i},D_{i}}^{2}+\sigma_{p_{1},D_{i}}^{2}$
$R_{i}$	($m_{D_{i},R_{i}}$, $\sigma_{D_{i},R_{i}}^{2}$)	$m_{R_{i},A_{i}}=m_{D_{i},R_{i}}$, $\sigma_{R_{i},A_{i}}^{2}=\sigma_{D_{i},R_{i}}^{2}$
$A_{i}$	($m_{R_{i},A_{i}}$,$\sigma_{R_{i},A_{i}}^{2}$), $\left(m_{y_{i},A_{i}},\sigma_{y_{i},A_{i}}^{2}\right)$,$\left(m_{z_{i},A_{i}},\sigma_{z_{i},A_{i}}^{2}\right)$	$m_{A_{i},x}=\left(1-k_{y,i}m_{y,A_{i}}-k_{z,i}m_{z,A_{i}}-k_{r,i}m_{R_{i},A_{i}}\right)/k_{x,i}$,
		$\sigma_{{}_{A_{i},x}}^{2}=\left(k_{y,i}^{2}\sigma_{y,A_{i}}^{2}+k_{z,i}^{2}\sigma_{z,A_{i}}^{2}+k_{r,i}^{2}\sigma_{R_{i},A_{i}}^{2}\right)/k_{x,i}^{2}$
		$m_{A_{i},y}=\left(1-k_{x,i}m_{x,A_{i}}-k_{z,i}m_{z,A_{i}}-k_{r,i}m_{R_{i},A_{i}}\right)/k_{y,i}$,
		$\sigma_{{}_{A_{i},y}}^{2}=\left(k_{x,i}^{2}\sigma_{x,A_{i}}^{2}+k_{z,i}^{2}\sigma_{z,A_{i}}^{2}+k_{r,i}^{2}\sigma_{R_{i},A_{i}}^{2}\right)/k_{y,i}^{2}$
		$m_{A_{i},z}=\left(1-k_{x,i}m_{x,A_{i}}-k_{y,i}m_{y,A_{i}}-k_{r,i}m_{R_{i},A_{i}}\right)/k_{z,i}$,
		$\sigma_{{}_{A_{i},z}}^{2}=\left(k_{x,i}^{2}\sigma_{x,A_{i}}^{2}+k_{y,i}^{2}\sigma_{y,A_{i}}^{2}+k_{r,i}^{2}\sigma_{R_{i},A_{i}}^{2}\right)/k_{z,i}^{2}$
*x*	$\left(m_{A_{i},x},\sigma_{{}_{A_{i},x}}^{2}\right)$	$m_{x}=\sigma_{x}^{2}\cdot \left(\sum_{i=1}^{n}\frac{m_{A_{i},x}}{\sigma_{A_{i},x}^{2}}\right)$, $\sigma_{x}^{2}=1/\left(\sum_{i=1}^{n}\frac{1}{\sigma_{A_{i},x}^{2}}\right)$
*y*	$\left(m_{A_{i},y},\sigma_{{}_{A_{i},y}}^{2}\right)$	$m_{y}=\sigma_{y}^{2}\cdot \left(\sum_{i=1}^{n}\frac{m_{A_{i},y}}{\sigma_{A_{i},y}^{2}}\right)$, $\sigma_{y}^{2}=1/\left(\sum_{i=1}^{n}\frac{1}{\sigma_{A_{i},y}^{2}}\right)$
*z*	$\left(m_{A_{i},z},\sigma_{{}_{A_{i},z}}^{2}\right)$	$m_{z}=\sigma_{z}^{2}\cdot \left(\sum_{i=1}^{n}\frac{m_{A_{i},z}}{\sigma_{A_{i},z}^{2}}\right)$, $\sigma_{z}^{2}=1/\left(\sum_{i=1}^{n}\frac{1}{\sigma_{A_{i},z}^{2}}\right)$

**Table 2:** Mean position error comparison with different grid distances.

Grid Distance	kNN	LS	Proposed Algorithm
1.5 m	1.41 m	1.37 m	1.12 m
2 m	1.62 m	1.46 m	1.25 m

**Table 3:** Mean position error comparison with different number of APs.

Number of APs	kNN	LS	Proposed Algorithm
3	1.83 m	1.58 m	1.41 m
4	1.65 m	1.37 m	1.15 m

## Abbreviations

The following abbreviations are used in this manuscript:

2-D	Two-dimensional
3-D	Three-dimensional
FG	Factor graph
kNN	k-nearest neighbors
LS	Least square
AP	Access point
TOA	Time of arrival
TDOA	Time difference of arrival
RSS	Received signal strength
ECLS	Extended candidate location set
RSSD	Received signal strength difference
SDP	semidefinite programming
LOS	Line of sight
NLOS	Non-line of sight
CDF	Cumulative distribution function
